# Navigating the adaptation of CBT for persons with disabilities in South Africa: An autoethnographic perspective

**DOI:** 10.4102/ajod.v15i0.1773

**Published:** 2026-04-24

**Authors:** Ramokone Kunutu, Sibusiso Ntshangase

**Affiliations:** 1 Department of Inclusive Education, College of Education, University of South Africa, Pretoria, South Africa; 2 Department of Psychology of Education, College of Education, University of South Africa, Pretoria, South Africa

**Keywords:** cognitive behaviour therapy, persons with disabilities, western-developed therapy, CBT, mental health

## Abstract

**Background:**

Cognitive behavioural therapy (CBT), a western-developed therapy, may offer benefits to persons with disabilities. However, its applicability in South Africa, where stigma and limited resources significantly impact mental health, is uncertain.

**Objectives:**

This autoethnography explores the first author’s personal experiences in adapting CBT for persons with disabilities in South Africa, focusing on culturally sensitive interventions and innovative delivery methods.

**Method:**

Spanning a year, data collection included personal journaling, field notes and reflexive memos. Rooted in constructivist epistemology, the study employed thematic analysis to integrate the first author’s personal narrative with the broader cultural context.

**Results:**

The study chronicles the discovery of innovative delivery methods, such as telehealth, and emphasises the critical importance of training local health professionals. Key findings address the evidence base for CBT, the successes and failures of adaptations, the knowledge gaps identified and promising directions for a more inclusive future.

**Conclusion:**

This autoethnography reflects on the path towards a mental health care system that effectively addresses the unique needs of persons with disabilities in South Africa.

**Contribution:**

The study provides insights into the adaptation of CBT for a specific cultural context, highlighting the importance of culturally sensitive interventions and innovative delivery methods to improve mental health care for persons with disabilities in resource-constrained settings.

## Introduction

I, the first author of this article, was first introduced to the challenges encountered by persons with disabilities^1^ in South Africa, not through a textbook, but during a chance encounter at a local disability awareness event in my hometown. I observed a young woman, perhaps in her early twenties, struggling to navigate the uneven terrain in her wheelchair. Her name was Thandiwe (pseudonym). She was attempting to reach the venue, but the entrance ramp was broken and overgrown with weeds. Her frustration was palpable, etched on her face as she repeatedly tried to manoeuvre her chair over the obstacle. Meanwhile, the organisers, seemingly oblivious, continued with their speeches inside. This encounter profoundly impacted me, leading me to recognise the systemic barriers, lack of accessible infrastructure, pervasive stigma and the absence of mental health support that trapped so many individuals in a cycle of suffering.

At the time, I was a psychology Honours student, volunteering at a local organisation that provided basic healthcare services to underserved communities. My mentor, who is listed as the second author of this article, invited me to attend a cognitive behavioural therapy (CBT) conference, where I was introduced to CBT and its potential to address various mental health issues. While Beck’s (2021) cognitive model, with its emphasis on the influence of thoughts and beliefs, initially appealed to me, a persistent concern remained regarding its applicability to the complex realities encountered by persons with disabilities in the South African context. This raised the question of whether a therapy developed within a western framework could adequately address the unique challenges and cultural nuances present in this specific community.

This initial scepticism was further reinforced by the stark realities revealed through literature review and statistical data. According to a recent report by the South African Depression and Anxiety Group (SADAG), persons with disabilities are three times more likely to experience depression and anxiety than the general population, yet only a fraction receive adequate mental health care (You et al. 2024). Recent statistics also indicate that persons with disabilities are significantly more likely to experience depression and anxiety compared to the general population (Wang et al. 2021; You et al. 2024). However, statistics alone fail to fully capture the lived experiences of individuals like Thandiwe. Recognising the limitations of relying solely on quantitative data, I understood the necessity of engaging directly with the community, listening to their narratives and gaining a deeper understanding of their specific needs. This realisation marked the beginning of my journey, one characterised by both hope and uncertainty, as I sought to bridge the barriers that separated persons with disabilities from the mental health care they deserved.

Historically, CBT has evolved through decades of research, demonstrating its efficacy across a range of psychological challenges and disorders. Its foundational theories, such as those proposed by Beck (2021), emphasise the role of cognitive processes in emotional regulation and behaviour change, making it particularly relevant for those facing the dual challenges of mental health issues and disability. Meta-analytic studies have provided support for the application of CBT across diverse populations, demonstrating its effectiveness in enhancing social skills and reducing anxiety in children and adolescents, including those with autism spectrum disorder (Sharma et al. 2021; You et al. 2024). However, despite this established efficacy, persons with disabilities in resource-constrained settings frequently encounter significant barriers to accessing mental health services, including pervasive stigma and a scarcity of adequately trained professionals (Naeem et al. 2023). To address these challenges, innovative delivery modalities, such as telehealth interventions and community-based programmes, offer the potential to improve accessibility and promote greater engagement (Rathod et al. 2017). Furthermore, the integration of culturally relevant adaptations and the leveraging of existing community support networks within CBT frameworks may contribute to improved outcomes and facilitate the development of a more inclusive and equitable mental health care system (Cucchi 2022; Hassinen & Lappalainen 2018).

Cognitive behavioural therapy has been recognised for its effectiveness across various mental health disorders, yet its applicability in diverse cultural contexts, particularly among persons with disabilities in South Africa, remains underexplored (Jalal, Kruger & Hinton 2020). The challenges faced by these individuals are compounded by systemic barriers such as stigma and limited access to mental health resources (Andersen, Rossouw & Kagee 2022). This highlights the urgent need for culturally sensitive adaptations of therapeutic interventions like CBT to better serve these marginalised groups.

Research has shown that culturally adapted CBT can enhance engagement and treatment outcomes among diverse populations, including indigenous groups in South Africa (Jalal et al. 2020). Recent studies emphasise the importance of integrating cultural elements into therapeutic frameworks to improve mental health accessibility and effectiveness. For example, Rosen et al. (2021) found that integrating psychosocial and economic support improved health outcomes for adolescents living with HIV in Zambia, underscoring the potential benefits of holistic approaches. Similarly, the systematic review by Seekles et al. (2023) highlights the effectiveness of preventive interventions tailored to the unique cultural contexts of adolescents in sub-Saharan Africa, suggesting that adaptations are crucial for successful implementation.

Despite the promise of the culturally adapted CBT, barriers to effective delivery remain. A review by Clay et al. (2020) identified core components of mental health stigma reduction interventions, emphasising the need for community engagement and training of local health workers. Mabunda et al. (2022) further demonstrated that interventions delivered by lay health workers can be culturally adapted to meet the needs of individuals with mental disorders, revealing a pathway for sustainable mental health care in resource-constrained settings. While the literature advocates for innovative delivery methods, such as telehealth, to improve accessibility, ongoing challenges related to resource availability and stigma necessitate continued research and adaptation efforts (McKenzie, Khenti & Williams 2024; Wallace, Carlson & Ohrt 2020).

Addressing these barriers will be essential for developing a more inclusive mental health care system that effectively serves persons with disabilities in South Africa.

The theoretical framework for this study was anchored in the social model of disability, which posits that disability is not simply a result of individual impairments but largely a product of societal structures and attitudes. The social model of disability emerged as a pivotal framework during the disability rights movement in the late 20th century, particularly in the United Kingdom (Dirth & Adams 2019). It arose in response to the dominant medical model, which primarily viewed disability as a deficiency or impairment located within the individual. In contrast, the social model of disability emphasised that disability resulted largely from societal barriers, discrimination and inadequate infrastructure that prevented individuals from fully participating in society. This theoretical framework was particularly relevant to this study, as it provided a critical framework for understanding and addressing the challenges faced by persons with disabilities. It reinforced the study’s focus on adapting CBT to promote empowerment, advocacy and systemic change in mental health care. By shifting the emphasis from individual impairments to societal structures, the social model of disability encourages a more inclusive approach to mental health interventions, ensuring that they are accessible and relevant to individuals with disabilities.

This autoethnographic article was motivated by a personal investigation into the effective adaptation of CBT for persons with disabilities within the South African context. It presents my experiences in critically examining the existing evidence base for CBT’s application to this specific population, questioning its relevance and applicability across cultural boundaries.

Furthermore, it investigates recent innovations in delivery modalities, such as telehealth, and documents my attempts to implement these strategies to enhance accessibility.

The article also elucidates the limitations and knowledge gaps encountered firsthand, revealing the complexities inherent in translating western theoretical frameworks into local practice.

Lastly, it highlights the promising directions that emerged from this work, informing a vision for future research and practice in this area.

The research questions were as follows:

- *What cultural, social and economic challenges do persons with disabilities face in accessing CBT in South Africa?*
- *How can established CBT frameworks be adapted to better align with the lived experiences and cultural contexts of persons with disabilities?*
- *What barriers do therapists encounter when implementing CBT for persons with disabilities, and how do these barriers affect treatment integrity and engagement?*
- *How do systemic factors, such as stigma and resource availability, influence the accessibility and effectiveness of CBT interventions for this population?*
- *What innovative delivery modalities, such as teletherapy and community-based programmes, can enhance access to CBT for persons with disabilities in resource-constrained settings?*

## Methods

### Research design

This article is derived from the data of a research project titled *Capacity Building for Disability Support*. It employs an autoethnographic approach that combines personal narrative with cultural analysis to explore my experiences in adapting CBT for persons with disabilities.

Rooted in constructivist epistemology, autoethnography acknowledges the researcher’s subjectivity as a valuable lens for understanding complex social phenomena (Creswell 2023).

This methodology facilitates a rich and nuanced exploration of the interplay between my personal journey and the broader cultural context of mental health care for persons with disabilities in a developing country setting.

### Participants

Within the framework of autoethnographic inquiry, the researcher assumes the pivotal role of primary data collection and analytical tool, drawing upon their lived experiences and introspective reflections as the foundational source of evidence (Goode, Lumsden & Bradford 2023). Although I occupy a central position, autoethnographic methodologies inherently acknowledge the significance of interactions with others, recognising their influence in shaping the narrative and providing essential contextual grounding for the process of self-exploration (Guyotte et al. 2018). Participants for this study can thus be delineated into two distinct categories, which are myself as the researcher, functioning as the primary participant, and the persons with whom I engaged and my mentor, designated as secondary participants.

#### The researcher

I was a Master’s student in psychology when this article was conceptualised. While I was doing my Honours degree, I volunteered in community projects supporting people with disabilities.

Through my mentor, I participated in initiatives focused on the adaptation and implementation of CBT for persons with disabilities. As a result, I gained a solid understanding of CBT principles as well as the specific challenges and opportunities presented by the South African socio-cultural landscape.

#### Secondary participants

The secondary participants in this study were 15 persons with disabilities, as well as my mentor, who had experience working with persons with disabilities. The experiences of these secondary participants were filtered through my subjective lens (Guyotte et al. 2018). While their voices were represented, the primary focus remains on my interpretation and analysis of these interactions. My mentor provided essential collaborative support and guidance throughout the research process.

#### Data analysis

I began by immersing myself in the data, reading and re-reading her journal entries, field notes and archival documents to gain a holistic understanding of her experiences. I then engaged in open coding, identifying key concepts, themes and patterns across the data. This involved assigning descriptive labels to segments of text that resonated with her research question. As I coded the data, I began to identify broader themes that captured the essence of my experiences.

These themes were refined and revised through ongoing analysis and reflection. Finally, I constructed a narrative that wove together her personal experiences with the broader cultural context, drawing on the identified themes to create a coherent and compelling story.

#### Rigour and trustworthiness

To ensure the rigour and trustworthiness of this study, several strategies were employed.

Prolonged engagement, involving sustained involvement in this work over a 1-year period, facilitated a deep and nuanced understanding of the complexities inherent in adapting CBT for persons with disabilities. Triangulation was achieved by corroborating findings through multiple data sources, including journal entries, field notes and archival documents, thereby enhancing the credibility of interpretations. Data were also shared with an experienced mentor, who is the second author of this article, to further enhance triangulation. Finally, reflexivity was maintained through a critical awareness of personal biases and assumptions throughout the research process, acknowledging the ways in which my subjectivity shaped the data and its interpretation, thereby strengthening the study’s overall trustworthiness.

#### Ethical considerations

Ethical clearance to conduct this study was obtained from the UNISA College of Education Ethics Review Committee (No. 2021/08/11/90129911/45/AM). This article emanated from data collected as part of a project, which had a blanket ethics clearance that permitted the exploration of different topics within community settings. Secondary participants provided informed consent before engaging in the study, and their confidentiality was rigorously maintained, with pseudonyms used in all reporting to protect their identities. Continuous reflection on ethical implications guided my interactions with participants, ensuring that their dignity and experiences were respected throughout the research process.

## Review findings

### The researcher’s evolving understanding of cognitive behavioural therapy’s effectiveness

The findings of this study highlighted the complex interplay between the established evidence base for CBT and the practical realities of adapting and implementing it for persons with disabilities in the South African context. While CBT demonstrates potential as a valuable intervention, the study revealed several key considerations. Firstly, direct translation of CBT approaches from western contexts to South Africa requires careful adaptation to account for the unique cultural, social and economic challenges faced by persons with disabilities. Culturally adapted CBT frameworks, aligned with the lived realities of persons with disabilities, are necessary for maximising the intervention’s impact (Naeem et al. 2023; Sharma et al. 2021). Examples from journal entries and field notes are as follows (the names used in the verbatim excerpts are pseudonyms I created for the purpose of this article):

**Cultural adaptation:**

‘Today I observed my mentor’s therapy session. He was using a standard CBT thought record, focusing on identifying negative automatic thoughts. The client struggled with the concept of isolating individual thoughts from the broader context of his family’s struggles with unemployment and access to basic services. The Western emphasis on individual cognitive processes felt almost … selfish, given his circumstances. My mentor needed to reframe the exercises to acknowledge the systemic issues impacting his mental well-being, by incorporating more culturally relevant metaphors and examples.’(Kunutu, journal entry, 29 June 2023)

**Economic challenges:**

‘Observed a significant barrier to homework completion among participants. Many reported difficulty accessing reliable transportation to attend sessions. One participant, Karabo, shared that the cost of transportation to the workshop venue consumed a significant portion of her disability grant, leaving limited resources for other essential needs. This highlights the need to integrate practical problem-solving strategies into CBT, such as budgeting and accessing community resources, to address these economic realities.’ (Kunutu, field note, 31 August 2023)

**Social stigma:**

‘During a group session … my mentor introduced the concept of assertiveness training, a common CBT technique. However, several participants expressed concerns about potential social repercussions within their communities. They feared being labeled as “difficult” or “disrespectful” if they asserted their needs, particularly when interacting with elders or authority figures. This underscores the importance of adapting assertiveness training to align with cultural norms and values, emphasizing respectful communication and conflict resolution strategies.’ (Kunutu, journal entry, 10 August 2023)

**Access to resources:**

‘We [*me and my mentor*] visited a rural community to conduct home-based CBT sessions. The lack of access to reliable internet connectivity posed a challenge for delivering telehealth services. Many participants relied on mobile data, which was often expensive and unreliable. This highlights the need for alternative delivery methods, such as printed materials and in-person sessions, to ensure equitable access to CBT for persons in remote areas.’ (Kunutu, field note, 14 September 2023)

Secondly, the findings indicated that despite evidence supporting CBT’s effectiveness in enhancing psychological well-being, anxiety reduction and improved coping strategies (Hassinen & Lappalainen 2018; Visagie et al. 2021), inequitable access to these interventions persists. Examples are as follows:

**Access barriers and transportation issues:**

‘During these sessions, we encountered a recurring theme: many participants expressed a desire to engage more deeply with CBT techniques but felt hindered by logistical barriers. For instance, Sipho mentioned that he could only attend sessions sporadically due to his work schedule, which often conflicts with clinic hours. This highlights the need for flexible scheduling or alternative delivery methods to ensure that persons like Sipho can access the support they need.’ (Kunutu, journal entry, 14 September 2023)

‘Today, I spoke with several participants about their experiences accessing CBT services. A common issue was transportation. Many persons come from far and rely on public transport, which is infrequent and often overcrowded. One participant, Lindiwe, shared that she missed her last two appointments because she could not afford the fare. This inequitable access to transportation directly impacts their ability to benefit from CBT, despite its proven effectiveness in enhancing psychological well-being.’ (Kunutu, field journal, 14 September 2023)

**Resource limitations:**

‘Visited a local clinic today to observe the delivery of CBT services. I noted that the clinic is severely understaffed, with only one psychologist available to serve a large population. Many persons seeking help are placed on long waiting lists, which can discourage them from pursuing treatment altogether. This inequitable access to qualified mental health professionals undermines the potential benefits of CBT, as timely intervention is crucial for effective anxiety reduction and coping strategy development.’ (Kunutu, field note, 20 July 2023)

Thirdly, in line with Benjamin et al. (2022), Coetzee et al. (2024), Mona, Hayward and Cameron (2019), Spreat and Roszkowski (2022) and Tapp et al. (2023), teletherapy and community-based programmes offer promising avenues for improving accessibility by overcoming geographic barriers, providing flexibility and fostering local support systems. Examples are as follows:

**Teletherapy success:**

‘Had a really encouraging teletherapy session with Zola today. She lives in a very remote rural area, and previously, it was impossible for her to attend in-person sessions due to the distance and cost of transportation. Through our video calls, she’s been able to consistently engage with the CBT exercises. She mentioned how much she appreciates being able to do the sessions from the comfort of her own home, without the stress of travel. She’s even started a small support group with other women in her village who are also using the teletherapy program. It’s amazing to see how technology, combined with community support, can break down barriers and make mental healthcare accessible to those who need it most.’ (Kunutu, journal entry, 28 September 2023)

### Navigating innovative applications of cognitive behavioural therapy

A key finding of this study is the inadequacy of directly transplanting western CBT models to the South African context, thereby underscoring the necessity of adaptation and innovation to ensure effective implementation. My personal experience underscores this tension, revealing a gap between theoretical recommendations and readily available, context-specific strategies.

The existing literature emphasises the importance of tailoring CBT to meet diverse needs, acknowledging the interplay of physical, cognitive and systemic challenges (Hassinen & Lappalainen 2018). The critical role of cultural adaptation is further highlighted, with Anderson et al. (2016) emphasising the need to integrate cultural elements into therapeutic frameworks. However, a key tension emerges regarding the practical implementation of these adaptations within resource-constrained environments, given infrastructural limitations (Rathod et al. 2017). For example:

**Adapting cognitive behavioural therapy protocols without losing core fidelity:**

‘[*During a conversation with my mentor he said*,] … I am struggling with the balance between providing standardized CBT protocols and tailoring the interventions to each individual’s unique needs. On one hand, I want to ensure fidelity to the evidence-based practices. On the other hand, I am seeing firsthand how rigid application of these protocols can be ineffective, even harmful, when they don’t align with a client’s cultural background or specific disability-related challenges. For example, I had a client with a severe visual impairment who was finding the standard CBT thought record worksheet completely inaccessible. I need to find ways to adapt these tools without compromising the core principles of CBT.’ (Kunutu, journal entry, 18 April 2023)

‘[*My mentor said*,] I had a frustrating day trying to implement a mindfulness exercise with a group of participants. The exercise required a quiet, comfortable space, but the clinic was overcrowded and noisy. It was impossible to create the calm environment needed for effective mindfulness practice. I realized that I need to be more creative and resourceful in adapting these exercises to the available resources. Perhaps we could try using guided imagery instead, or find a quiet space outdoors.’ (Kunutu, journal entry, 18 April 2023)

While innovative applications such as relaxation training, parental involvement and biofeedback (Tapp et al. 2023; Tassé et al. 2022; You et al. 2024) demonstrated promise, my engagement with the literature and practice reveals a persistent challenge in identifying concrete examples of effectively integrating culturally responsive strategies despite their acknowledged importance.

Findings on the application of CBT for older persons with disabilities, a demographic often facing unique challenges, revealed that CBT has the potential to be a safe and effective treatment option, with minimal side effects compared to pharmacological interventions. In line with Graser et al. (2022), the benefits of CBT and group therapy, as well as the importance of community-based interventions that incorporate culturally sensitive adaptations (Rathod et al. 2017), were also highlighted. For example:

**Cognitive behavioural therapy as a safe, empowering tool:**

‘Today, I observed a session with Ms Kganyago, a woman with mobility challenges. We focused on cognitive restructuring techniques to help her manage her anxiety about falling. She expressed relief at being able to discuss her fears in a safe space without the side effects associated with medications she had previously tried. It was heartening to see her engage with the CBT exercises, and she reported feeling more empowered and in control of her thoughts. This reinforces my belief that CBT can be a safe and effective treatment option for older adults, especially when tailored to their specific needs.’ (Kunutu, field journal, 10 August 2023)

A challenge highlighted by the findings was the issue of low literacy rates among rural populations with disabilities. This underlined the inaccessibility of traditional CBT methods, which often rely on written materials, for this demographic. The study revealed that innovative approaches, such as the use of visual aids, storytelling and community-based workshops (Sharma et al. 2021), may be useful regardless of the challenges related to low literacy.

Finally, the study’s investigation into the potential of teletherapy and mobile health technologies to bridge the gap in mental health services in rural areas suggests that these technologies may be particularly effective when combined with community resources and support networks (Spain & Happé 2020; Tapp et al. 2023; Wieland et al. 2024). My personal exploration highlighted the potential of these technologies while also acknowledging the need for careful consideration of access to technology and digital literacy within these communities.

### Addressing access barriers and unveiling gaps in research and practice

The findings of this study revealed gaps in both research and practice regarding CBT for persons with disabilities in South Africa. One of the primary gaps identified was the lack of knowledge concerning optimal treatment content, format and dosage. As highlighted by Naeem et al. (2023), there were no standardised treatment manuals or clear guidelines that specified the most effective elements of CBT interventions for this population. This absence made it challenging for practitioners to deliver consistent and effective treatment.

Additionally, the quality of research surrounding CBT for persons with disabilities raised serious concerns. According to Wieland et al. (2024), the variability in trial and treatment quality was problematic, as there were no standardised measures across different studies. This inconsistency complicated the ability to compare results and draw meaningful conclusions about the efficacy of various interventions.

Another critical gap pertained to treatment integrity, particularly regarding therapist competence. The study underscored the insufficient research on the qualifications and skills necessary for therapists delivering CBT to persons with disabilities. This lack of focus on therapist competence raised questions about the integrity of the treatment being provided and its potential impact on patient outcomes.

Moreover, the challenge of participant engagement in treatment emerged as a concern. It was noted that fostering active participation among persons with disabilities went beyond mere attendance at sessions. Engaging these individuals in the therapeutic process and encouraging them to apply learned skills were crucial for successful outcomes, yet this remained a major hurdle.

The study also pointed to a limited understanding of the mechanisms that underpinned treatment effectiveness. There was a notable gap in knowledge regarding the cognitive and behavioural processes that mediated the effects of CBT on outcomes, especially within disabled populations. Furthermore, there was a lack of insight into patient characteristics that may have served as moderators affecting the success of CBT interventions.

Finally, perhaps the most pressing gap identified was in the translation of research findings into practice. The study highlighted the need for effective strategies to facilitate the widespread adoption of evidence-based CBT interventions tailored for persons with disabilities in South Africa. This required a focus on understanding consumer needs and fostering community engagement to bridge the gap between research and real-world application.

## Implications and recommendations

The gaps identified in the research and practice of CBT for persons with disabilities in South Africa carry several critical implications for the field.

Firstly, the lack of standardised treatment manuals and clear guidelines on optimal content, format and dosage hampers practitioners’ ability to deliver effective interventions. Without a solid foundation of best practices, therapists may struggle to provide consistent care, leading to variable outcomes for clients. This inconsistency could result in a loss of trust in therapeutic processes among persons with disabilities, making them less likely to engage with available mental health services.

Secondly, the concerns around the quality of research indicate a pressing need for the development of standardised measures across trials. The current variability makes it difficult to compare results, undermining the validity of findings and limiting the potential for evidence-based practice. This lack of comparability could slow the advancement of effective CBT interventions, delaying the benefits that could be derived from robust research.

The gap in understanding treatment integrity, particularly regarding therapist competence, suggests a need for targeted training and professional development. Without a focus on enhancing therapist skills, the effectiveness of CBT for persons with disabilities may be compromised. This could perpetuate a cycle of inadequate treatment, where clients do not receive the quality of care they need, further exacerbating their mental health challenges.

Moreover, the challenges related to participant engagement highlight the necessity for innovative strategies to foster active involvement in therapy. Without addressing this barrier, the effectiveness of CBT interventions is likely to remain limited. Engagement goes beyond attendance; it requires therapists to create an inclusive environment that empowers clients to actively participate and apply their learning. Failure to enhance engagement could result in poor therapeutic outcomes and client dissatisfaction.

The limited understanding of the cognitive and behavioural mechanisms underlying treatment effectiveness points to a significant area for future research. A deeper investigation into these mechanisms is essential to tailor interventions to meet the unique needs of disabled populations. Without this understanding, interventions may remain generic and fail to address the specific challenges faced by these individuals.

Lastly, the gap in translating research findings into practice underscores the importance of effective knowledge transfer strategies. Bridging this gap is crucial for ensuring that evidence-based CBT interventions reach those who need them most. This requires collaboration between researchers, practitioners and communities to develop accessible resources and training that align with the needs of persons with disabilities. If left unaddressed, this gap could hinder progress towards achieving equitable mental health care for all individuals.

### Strategies for mitigating identified gaps in research and practice

Addressing the gaps in research and practice concerning CBT for persons with disabilities in South Africa requires a multifaceted approach. Collaborative efforts among stakeholders, including researchers, clinicians and individuals with lived experiences, are essential to develop standardised treatment protocols that provide clear guidelines on treatment content, format and dosage. This collaboration will help ensure that practitioners can deliver consistent and effective interventions tailored to the unique needs of this population.

Improving the quality of research is another critical strategy. Adopting standardised measures and methodologies across studies can enhance comparability and reliability, allowing for more robust evaluations of treatment efficacy. Additionally, fostering partnerships between researchers and practitioners can promote the exchange of best practices and support ongoing professional development for therapists. Innovative engagement strategies, such as using technology and involving family members in the therapeutic process, can further enhance participant engagement, encouraging active participation and skill application.

Effective knowledge translation and advocacy for policy change are vital for bridging the gap between research and practice. Developing accessible resources and training programmes can help practitioners implement evidence-based interventions, while collaboration with community organisations ensures that these resources meet the needs of the target population. Advocating for supportive policies will further enhance access to quality mental health care for persons with disabilities. By implementing these comprehensive strategies, the field can move towards more effective, inclusive and equitable mental health care, ultimately improving the well-being of persons with disabilities.

## Conclusion

This autoethnography concludes that while CBT holds potential for persons with disabilities in South Africa, its effectiveness is not guaranteed and requires careful adaptation. Tailored interventions, integration into primary care and innovative delivery methods like telehealth are crucial. We recommend the need for ongoing evaluation and quality improvement while acknowledging the limitations of the study’s generalisability due to its personal nature. This article aims to inspire further research and action in this area.

As an autoethnography, this article is inherently constrained by its focus on my personal experiences. While efforts have been made to provide a nuanced and contextualised account, the perspective presented is necessarily shaped by my background, values and beliefs.

Furthermore, the findings may not be generalisable to other contexts or populations. Nevertheless, it is anticipated that this autoethnography will offer valuable insights into the challenges and opportunities associated with adapting CBT for persons with disabilities in a resource-constrained setting and that it will stimulate further research and action in this domain.

## Footnotes

1 The term ‘persons with disabilities’ is used in this article, as it is favoured by many scholars in the field of disability studies, including Ohajunwa, Dube and Chitando (eds. 2025).
